# Practical Guide to Automated TEM Image Analysis for Increased Accuracy and Precision in the Measurement of Particle Size and Morphology

**DOI:** 10.1021/acsnanoscienceau.4c00076

**Published:** 2025-04-17

**Authors:** Kristen M. Aviles, Benjamin J. Lear

**Affiliations:** Department of Chemistry, 311285 The Pennsylvania State University, University Park, Pennsylvania 16802, United States

**Keywords:** Nanoparticle, transmission electron microscopy, FIJI, imaging, AI, image analysis, statistics, data visualization

## Abstract

A common desire in nanoscience is to describe the size and morphology of nanoparticles as observed from TEM images. Many times, this analysis is done manually, a lengthy process that is prone to errors and ambiguity in the measurements. While several research groups have reported excellent advances in machine-learned approaches to automated TEM image processing, the tools that they have developed often require specialized software or significant knowledge of coding. This state of affairs means that a majority of researchers in the field of nanoscience are not well-equipped to incorporate these advances into their normal workflows. In this tutorial, we describe how to use Weka segmentation within the free and open source program FIJI to automatically identify and characterize nanoparticles from TEM images. The approach we outline is not meant to discount the excellent results of groups working at the forefront of machine learning image analysis; rather, it is meant to bring similar tools to a broader audience by demonstrating how such processing can be done within the GUI-based interface of FIJI—a program already commonly used within nanoscience research. We also discuss the advantages that arise from automatic processing of TEM images, including repeatability, time savings, the ability to process low-contrast images, and the additional types of characterization that can be performed following identification of particles. The overall goal is to provide an accessible tool that enables a more robust and repeatable analysis and descriptions of nanoparticles.

## Graphical abstract

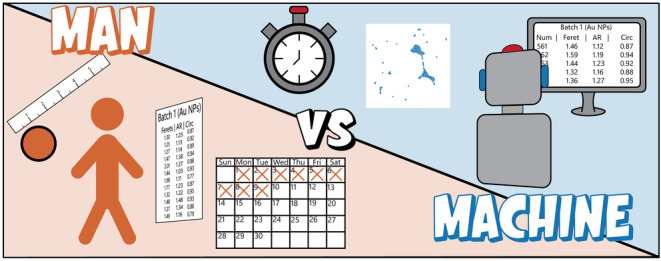

## 1 Introduction

A hallmark of nanoparticle research is that size and morphology control the behavior. This is true across diverse areas of study and application, including electronic structure,^1–7^ absorption,^8,9^ emission,^8,10^ scattering,^9^ catalysis,^11,12^ and clearance in biological systems.^9,13^ For this reason, determining the size and morphology of nanoparticles is an integral aspect of research involving them.

Though there are numerous means by which to determine particle size and morphology, the analysis of transmission electron microscopy (TEM) images currently dominates the field.^14,15^ Figure 1A,B shows two examples of TEM micrographs that might be reasonably encountered during nanoscience research. An advantage of using imaging is that one is shown the particle’s shape—albeit a 2D projection—so size and morphology are directly accessible. However, this approach also carries with it at least three disadvantages regarding measurement, reproducibility, and throughput. This tutorial explores and proposes solutions to these challenges, based on the automated processing of images using machine learned Weka segmentation^16,17^ implemented in the free open-source program FIJI.^18^

**Figure 1:**
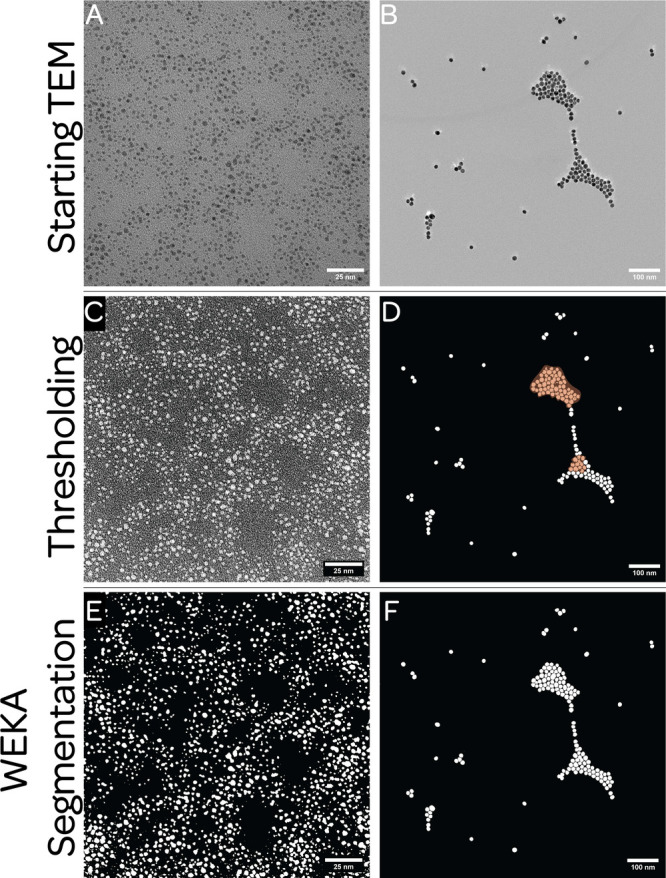
Examples of TEM migrographs that might be used to characterize nanoparticles. (A) A TEM migrograph with relatively low contrast. (B) A TEM micrograph with relatively good contrast. (C, D) Results of processing the original micrographs using thresholding. (E, F) Processing of the original micrographs using Weka segmentation.

Before moving on, it is worth explicitly acknowledging that addressing this problem is an area of active research. Specifically, many research groups are producing excellent results in the area of machine learning and automated image processing.^19–24^ The solution proposed below is not intended to downplay these efforts or solutions. However, we also acknowledge that cutting-edge tools often require specialized software or significant programming expertise to utilize. The approach outlined below was chosen because it is available, accessible, free, and implemented in software that is already widely used by the nanoscience research community. While emerging tools may offer greater efficiency, accuracy, or capabilities, the approach we outline below can provide a significant step to ameliorating the effects of the three problems introduced above, on which we now expand.

### 1.1 Ambiguity in Measurement

To begin with, there is scant guidance regarding how to measure the desired aspects of particles.^25–27^ For instance, consider the particles shown in Figure 1 and the ambiguity associated with even the ubiquitously reported particle “size”. Is the size the longest chord across a particle? The mean chord? The median chord? Would the area be a better measurement of size? While many articles report “size”, this quantity is ill-defined for all but the most regular of particles. This problem is compounded when one wishes to discuss other features like aspect ratio (which requires the measurement of at least two “sizes”) or shape (which could require measurement of many “sizes”).

### 1.2 Issues of Reproducibility

Herein we define reproducibility as the ability to duplicate results when performing the same workflow on the same materials as a different researcher. The issue of reproducibility in part stems from the ambiguity in the measurement discussed above. Two scientists measuring the “size” of particles by hand will almost certainly produce different results, which can quite easily result from systematic differences between the scientists’ treatments. Even repeatability (which we define as the ability of a single researcher to duplicate their results) is a challenge, as a single researcher could quite easily produce different results by analyzing the same image twice. For instance, variation in fatigue may lead to variations during the analysis. Ignoring both ambiguity in measurement and variation in attention, there can be challenges in identifying particles of interest or the boundaries of these particles. While particle identification might prove straightforward for images with high contrast, such as that seen in Figure 1B, it is routine to encounter TEM images with low contrast (e.g., Figure 1A) where judgment calls must be made about what is a particle and where the boundaries of these particles lie. Even when strong contrast is present, it can prove challenging to consistently identify the edges of the particles.

### 1.3 Very Low Throughput

The ambiguities involved in manual processing of TEM images lead us to the final problem considered here: the requirement for repeated judgment calls in analyzing an image, combined with the fact that attaining statistically meaningful data requires hundreds of such measurements, leads to a severe bottleneck in image processing that severely limits research throughput. A standard rule of thumb in the field is that attaining statistically meaningful estimates of particle properties often requires measurement of at least 200 particles.^28^ In our lab, experienced researchers might be able to size that many particles with good contrast (i.e., Figure 1b) in an hour. This suggest that if the entirety of a typical work week were devoted to analyzing TEM images, at most 40 samples could be characterized in that week. The number of samples that can be processed decreases as the image contrast is reduced, new aspects of particles are introduced, and the number of other activities performed by the researcher (writing, synthesis, group meetings, etc.) increases. In addition, we note that the analysis of particles is expected to improve as the number of particles increases. This is illustrated in Figure S9. Thus, the standard suggestion of counting 200 particles is a result of a compromise between the desire for accurate analysis and the time involved in measuring particles. In other words, low throughput also places a limit on the accuracy of the analysis within a single sample.

## 2 A Solution

All of the above considerations could be addressed by automated processing of TEM images by computer software. Automation obviously addresses the throughput issue, but it also has the potential to address both reproducibility and measurement ambiguities. By processing images algorithmically, one can reduce the fluctuation between users and also create a situation where analysis is effectively perfectly repeatable for a single user. Additionally, the image processing can identify the bounds of particles, thereby allowing consistent identification of the particles and a means by which to clearly define characteristics such as size and morphology.

Below, we demonstrate one approach to the automatic processing of TEM images. The solution we outline uses a free plug-in for the free program FIJI. Thus, it relies only on a graphical user interface within a program that is already familiar to many in the field. Below, we demonstrate how this program can be used to (1) automatically identify particles and (2) perform consistent and meaningful measurements on the identified particles. We then consider how one might report the results of these measurements and then use these reports to demonstrate the advantages of this approach among five researchers. It is our hope that introduction of this tool will both alleviate the challenges outlined above for many researchers and lead to more consistent and meaningful reporting of nanoparticle size and morphology.

### 2.1 Challenges Associated with Simple Thresholding

Identification of particles in images relies upon contrast between the particle and the background.^19,20,27^ Thus, all approaches to automating the identification of particles somehow seek to leverage these contrast differences. We note that the discussion that follows focuses on TEM, in which particles are typically presented as dark against a lighter background, though the same general approach holds true for other particle measurements like grayscale scanning electron microscopy (typically white particles against a dark background) or even full-color optical microscope images.

The most straightforward approach to procedurally distinguish particles from the background is through thresholding. To threshold the TEM images shown in Figure 1A,B, a gray value is chosen that is between that of the darker particles and the lighter background. Grays that are darker than this threshold value are assigned as particles, while lighter grays are considered background. Often, the image is then recolored based on this threshold: with one side being colored white and one side being colored black. FIJI supplies the capabilities to accomplish this, and one can find multiple tutorials on this approach, though (as discussed next) we do not recommend the use of simple thresholding.

Figure 1C,D shows the results of such thresholding applied to the original images. In examining the resulting images, it is immediately clear that thresholding is much more effective when one is working with an already high-contrast image. It is not clear that the task of particle identification has been improved for the low-contrast image—and it may have been complicated, as there are numerous places where very small white regions now exist. If this image were to be further processed by a computer using the threshold as a delineator for particles, then the results would contain many spurious particles. Thus, it is clear that thresholding is a fraught process for low-contrast images. However, even for the high-contrast images, thresholding is not perfect. Looking at the regions highlighted in orange in Figure 1D, one can see that particles have been missed or portions of particles have been missed. The need to address these areas reintroduces ambiguity and reproducibility issues while lowering throughput.

### 2.2 Consistent Automatic Identification of Nanoparticles Using Machine Learning: Trainable Weka Segmentation

Machine learning tools can overcome the difficulties in particle identification for both low-contrast and high-contrast images. These tools do not use strict thresholding but instead are able to “learn” the *patterns* of gray associated with either particles or background.

Within FIJI, the Trainable Weka Segmentation plugin for FIJI provides a ready solution. To navigate to this plugin, select Plugins > Segmentation > Trainable Weka Segmentation. A generalized workflow requires six broad steps, as shown in Figure 2. A detailed step-by-step walkthrough moving through each of these steps can be found in the Supporting Information (SI) of this tutorial, and the data needed to reproduce the results presented in this tutorial are linked in the SI. When using this plugin, one first creates “classes” (e.g., mesh and particle), and then one identifies one or more regions in an image that correspond to each class. The freehand selection tool is useful for identifying large areas. When an area is selected, it can be assigned to the appropriate class (Add to Class). Once sufficient samples for classes have been identified, the model can be trained on these samples (Train Classifier). If the training does not provide the desired segmentation, more regions can be assigned to each class and the model retrained, though the time needed to perform the training will increase as more regions are added. Thus, there exists a tension between attaining the desired results and the time required to do so. In our experience, it is more efficient to start with a small number of regions and then add more, when needed rather than to start with a large number of regions.

**Figure 2:**
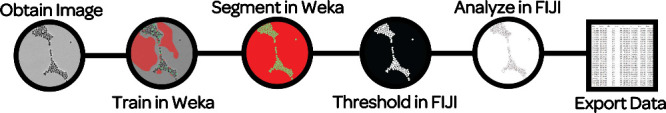
Workflow was used for image processing. We first begin by obtaining the microscopy image. The image is then trained in Weka. Once training is sufficient, the image is segmented, affording a red-and-green output. Then the red-and-green images are opened in FIJI by selecting Create Result and thresholded. Analysis is performed via FIJI’s Analyze Particles capability. Finally, the results are exported as a .csv file, allowing further workup of the data.

After verification that the classifier provides the desired behavior (see the SI), it can be saved. For images of similar contrast, the classifier can be applied directly by selecting the image and selecting Apply Classifier. This will automatically segment and open the image in FIJI using a green-and-red binary scheme, which can be converted to the binary threshold for further analysis, similar to what could be done following basic thresholding. To use this function, the image must not be a .ser or .emi file type.

Figure 1E,F shows the results of applying a trained Weka segmentation to the original TEM images (Figure 1A,B). Examining these images, it is immediately clear that the Weka segmentation has provided better identification of the particles for both the low-contrast and high-contrast images.

Close examination of the segmented images may still reveal regions where clusters of particles have not been separated or extremely small objects (which are likely not particles) have been identified as particles. The presence of these objects can be handled in one of three ways. First, the regions of error could be added to the training so that the final model will handle them better. Second, one can set lower and upper limits for particle size in FIJI, which results in features that are too large or too small being automatically excluded from the subsequent analysis. Finally, each feature identified is assigned an ID (see the SI), and one can manually remove errors by ID. In our experience, some combination of the first and second approaches yields excellent results while avoiding manual processing.

The most time-intensive component for this segmentation is the initial training of the model, and the training time depends on the exact application of processing (filters, blurs, etc.) and the image file size. However, for many TEM images, the training can be accomplished in tens of minutes on a standard laptop. For the low-contrast TEM image (Figure 1A), training the model took 20 min on a 2022 model Microsoft Surface, while the training for the high-contrast image took 7 min. After the training of the model, application to further images is similarly quick—taking just a few minutes on this same Microsoft Surface. Thus, when multiple images are to be processed, the use of Weka segmentation offers *substantial* time savings over manual particle identification—which only increases as the number of processed images increases. Finally, while the training of the model does require human input, the application of this trained model does not. Thus, at least at the level of identifying individual particles, human bias and shortcomings are removed.

### 2.3 Measurement of Particle Properties

A particular advantage of using automated nanoparticle identification is that the segmented image can be used to attain the outlines of the particles (Figure 3A). This in turn provides an opportunity to perform programmatic analysis on the particles—further removing human bias and error from extracting size and morphology information on the particles.

**Figure 3:**
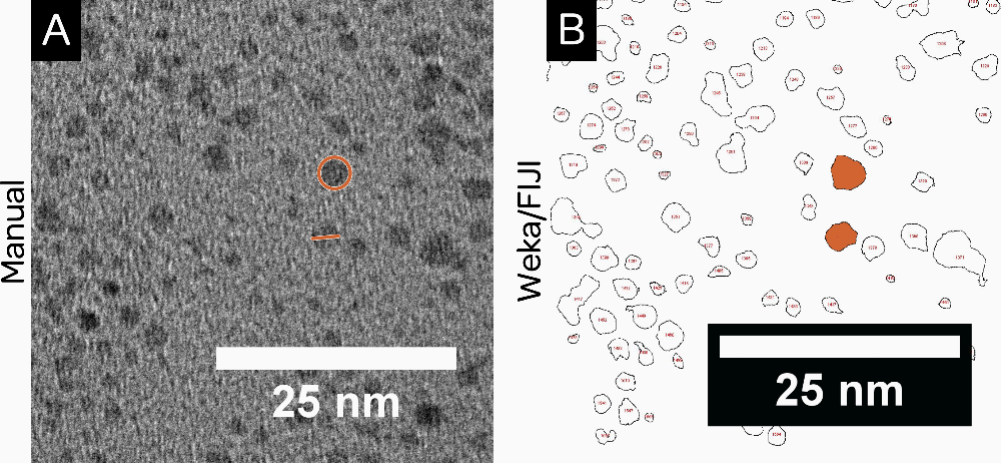
Comparison of (A) a region of the low-contrast TEM image (Figure 1A) and (B) the outlines of particles identified in this same region by Weka segmentation. Shown in (A) are examples of how size measurements might be made by hand (orange markings), which involve human judgment as to where the edges of the particles are. The outlines obtained in (B) remove this ambiguity.

To understand how this is the case, let us return to the idea of “size”. If one works with the raw image, sizing often involves either drawing a line across the imagined face of the particle or drawing an ellipse around the particle (Figure 3B). However, both of these involve guessing at where the edges of the particle lie and still begs the question of which line should be drawn or what component of the ellipse should be measured.

On the other hand, with an outline of the particle attained after segmentation (Figure 3B), rigorously defined measurements can easily be made. For instance, if one believes that the particles being measured are circular in nature, then one can simply solve for the “effective” diameter (*d*) of the particle derived from the area (*A*): $d=\sqrt{A/\pi }$ . Likewise, if one believes the particles are squares, then a reasonable value of the size might be simple: $d=\sqrt{A}$ . Similar values can be arrived at for other shapes, or one could base them off of the value of the length of the outline. In this case, circular particles could have their diameter estimated from the circumference (*c*): *d* = *c*/π.

The above values are convenient because they harness the directly measured area of the particles. Additionally, they provide a sort of “averaging” across the irregularities present in real particles. However, one problem is that one must make assumptions about the underlying shape, and the accuracy of the extracted values will be directly related to how well the real particle approximates this assumed shape. Additionally, the interpretation of the value differs. Above, the definition for the circular systems is associated with the longest chord, while for the square it is the length of a side and not the longest line through the object (i.e., the diagonal line).

A measurement of size that avoids both of these complications is the so-called Feret diameter. This is defined as the distance between two parallel lines running tangential to the particle, and the maximum value for three prototypical particle shapes is illustrated by the dashed lines in Figure 4. This measurement simulates the more familiar measurement of objects using calipers, so the Feret diameter is also known as the “caliper diameter”. For any given outline, there will be both minimum and maximum Feret diameters, which can be uniquely identified in a consistent and algorithmic manner. Importantly, both values are present (though they may be identical) and equally valid, no matter the shape of the particle. Thus, when discussing size, reporting either (or both) of these values provides a reproducible and nonambiguous means by which to discuss their size—though it is worth noting it does not have the sort of “averaging” effects that are produced for the values derived from area considerations.

**Figure 4:**
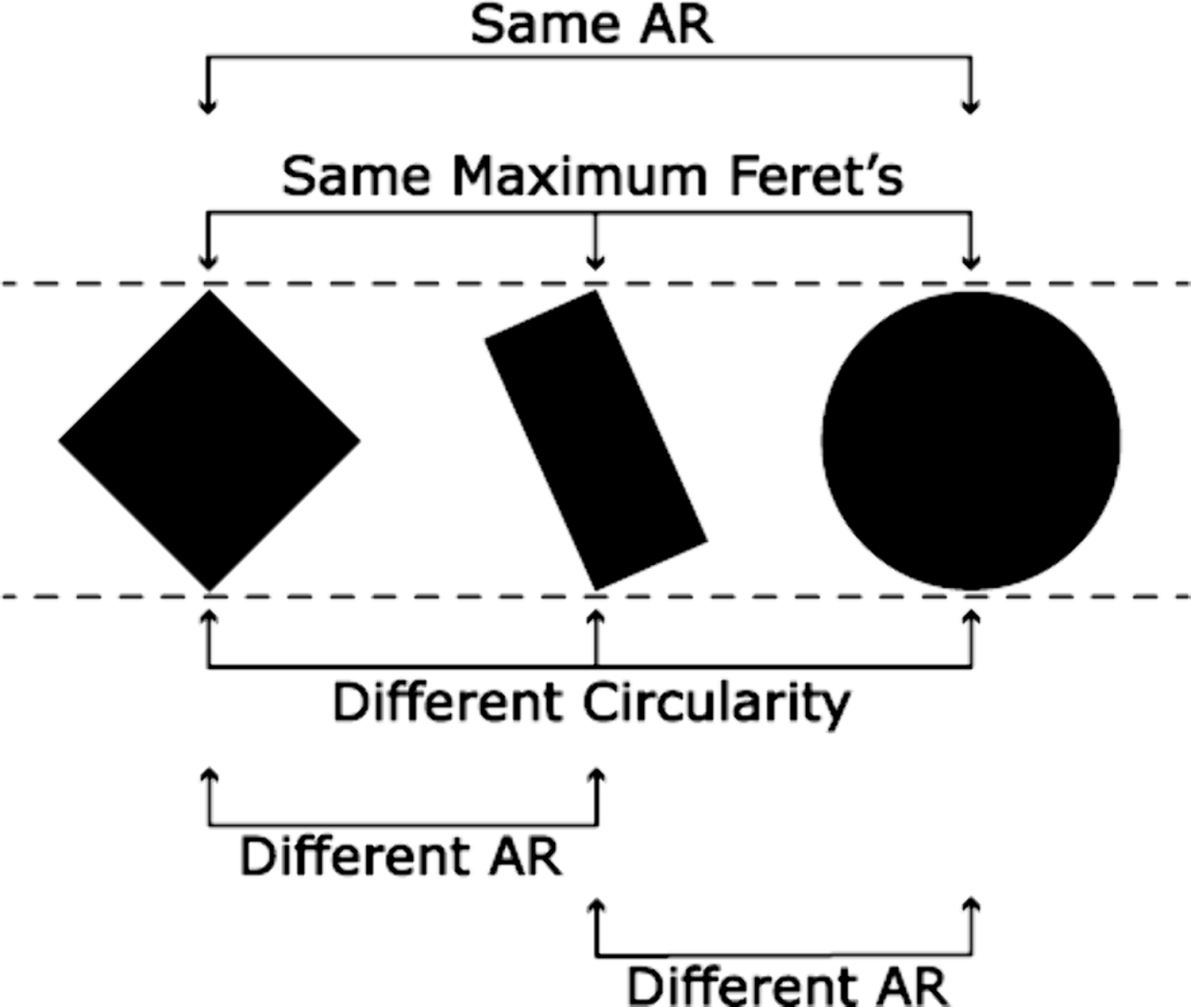
Illustration of a few of the measurements that can be made on particles identified by segmentation. Size can be measured using the Feret diameter, while aspect ratio (AR) and circularity speak to morphological considerations.

Beyond size, there are many other measurements that could be of interest and which speak to morphology. Perhaps the most often used is the aspect ratio (AR). The most common approach within the literature appears to be accomplished by drawing lines along what are perceived as the long and short axes of particles. However, this approach again brings all of the ambiguities associated with measurements performed by hand. Alternatively, one can have FIJI fit an ellipse to the outline of the particle and then use the ratio of the ellipse’s major axis to its minor axes as the measure of AR:

$$\mathrm{AR}=\frac{\mathrm{Major}\;\mathrm{Axis}}{\mathrm{Minor}\;\mathrm{Axis}}$$ 1

This approach again removes human error and leads to greater reproducibility in the measurement. There are also other possible measures of AR (for instance, calculating moments of inertia for the area of the particle), but these are not readily available within FIJI so we do not consider them further.

While AR is widely used to describe the morphology of particles, there are other measurements that might be of use. For instance, even though a human can easily distinguish between the circle and square drawn in Figure 4, both of these shapes have the same maximum Feret diameter and AR.

One option to distinguish between these shapes is to report both the maximum and minimum Feret diameter. Another method is to use the metric of circularity. It is important to note that the terms “circularity” and “roundness” are often used interchangeably. From the position of ASTM Standard F1877-16,^25^ roundness is used exclusively, but the definition matches that for circularity used within FIJI. In FIJI, circularity (ASTM roundness) is defined as the ratio of the area to the square of the perimeter of a particle, all scaled by 4π:

$$\mathrm{Circularity}=\frac{\mathrm{Area}}{{\mathrm{Perimeter}}^{2}}\cdot 4\pi$$ 2

In this definition, the value of circularity ranges from 1 (a circle) to 0.^29^ The use of circularity allows for distinction between all three objects in Figure 4 while also having the benefit that it can be programmatically calculated from the particle outlines obtained by segmentation. Circularity (ASTM roundness) also has the benefit that familiar shapes have set values: the roundness of a circle is always 1, the roundness of a square is always $1/\sqrt{2}$ , and so on. Of course, some shapes, such as scalene triangles, may have a large range of possible values for circularity. The point of this discussion is not to provide an exhaustive resource on how to parametrize and differentiate morphology but to point out that FIJI provides access to tools that have utility in this area—tools that would be difficult to apply without access to particle outlines, as Weka segmentation provides.

We conclude this section by noting that the outlines of the particles within FIJI could be exported for use in external programs to enable an even more sophisticated analysis. For instance, FIJI does not report the uncertainties associated with the major and minor axes obtained by fitting an ellipse to particles, but such uncertainties could be valuable to know. One could readily produce a Python script to find and report these values. Alternatively, further processing of the outlines may be pursued to decrease the pixelated boundary for what is assumed to be a circular or spherical particle. This may be performed, through smoothing functions but can be a misrepresentation of the data due to image manipulation. Thus, smoothing should be executed only if supported by scientific reasoning. Nevertheless, even working entirely within FIJI affords a more reproducible and holistic expression of the true shape of the particle and allows differentiation of more particles morphologies than could be obtained solely via manual processing.

## 3 Reporting of Nanoparticle Properties

Beyond tunability, another hallmark of nanoparticles is their heterogeneity. Outside of a very small subset of atomically precise clusters/particles,^30^ all nanoparticles contain some amount of structural heterogeneity. This means that when reporting on the size or morphology of nanoparticles, we are actually reporting on a *population* of these properties, and it is worth addressing how best to report the distribution of particles in these populations.

The histogram has long served as a staple of reporting distributions and is well-served for reporting nanoparticle distributions. Figure 5 shows examples of histograms for the maximum Feret diameter, AR, and circularity of the particles shown in Figure 1. A histogram is produced when a *continuous* variable is binned into discrete regions; in this case, the *x* axis is the continuous variable. For this reason, the bars along this axis should be touching, as gaps between the bars would represent areas where data were not binned or where no observations were made. The SI has a discussion of how the generation of a histogram may be done, for those unfamiliar with doing so.

**Figure 5:**
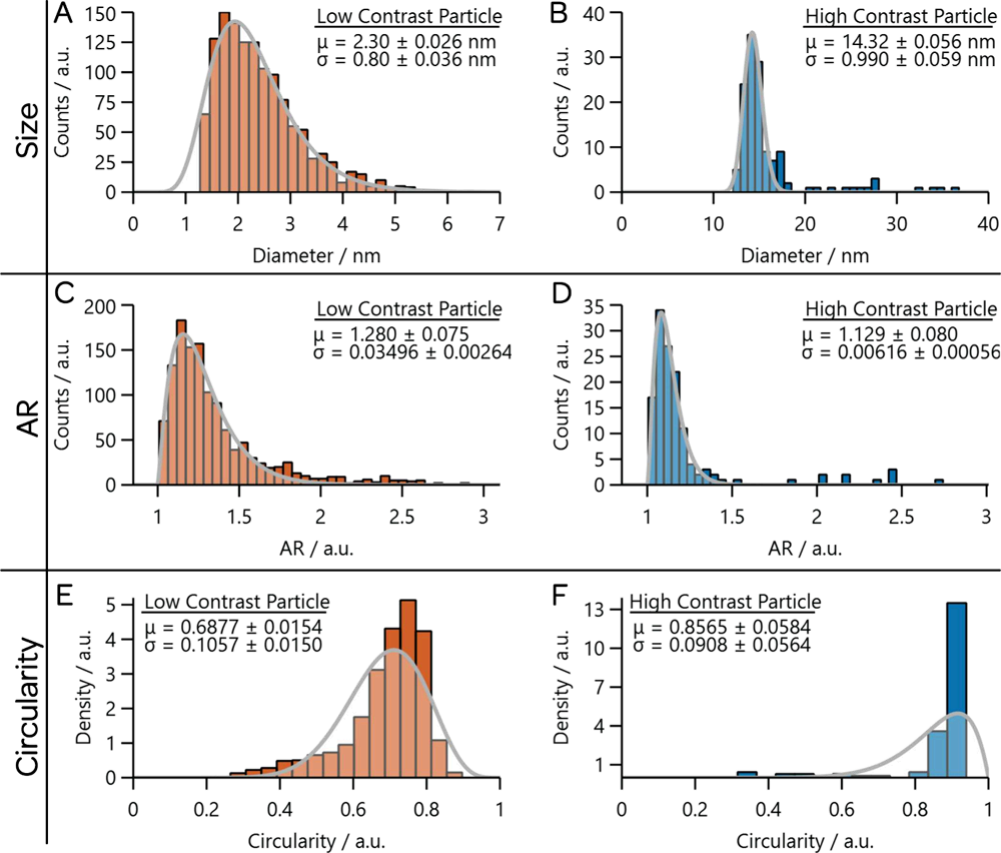
Histograms associated with measurements of particles in the TEM images shown in Figure 1. The left column is associated with the low-contrast images, while the right column is associated with the high-contrast images. The top row shows the histograms of maximum Feret diameter and the fit of a log-normal distribution to the data. The middle row shows the histogram for AR and the fit of the gamma distribution to the data. The bottom row shows the histogram for circularity and the fit of a beta distribution to this data. In each case, the extracted mean and standard deviation from the fit are provided, as are the uncertainties in these values estimated from the fit.

While the histograms shown in Figure 5 represent the overall shape of the distribution, summary statistics are often of interest, with the mean (μ) and standard deviation (σ) of the measurement being the most often presented. It can be tempting to report the arithmetic mean and standard deviation for the particles; however, it is worth noting that these numbers can easily be quite misleading.

Consider, for instance, the histogram of size for the smaller particles or the histogram of circularity for the larger particles. Both of these display a clear skew in their distributions. Thus, the arithmetic mean will not represent the center of the distribution, while the standard deviation will likely be overestimated due to the values found at one extreme of the distribution.

A better approach to finding means and standard deviations is to identify the distribution that is associated with the population. For nanoparticle size, it is well-known that the population follows the log-normal distribution:^31,32^

$$f\left(x;\mu ,\sigma \right)=\frac{1}{x\sigma \sqrt{2\pi }}\exp\left[-\frac{{\left(\ln x-\mu \right)}^{2}}{2\sigma^{2}}\right]$$ 3

where *x* is the measured size, μ is the mean particle size for the distribution, and σ is the standard deviation of this distribution. Thus, we can fit this distribution to the data in the histogram and then extract the mean and standard deviation from the fit. It is worth noting that these are the mean and standard deviation associated with the lognormal distribution, and are not the “real-space” (i.e., nanometers) parameters. However, the “real-space” parameters can be obtained from the lognormal parameters, as outlined in the SI. It is also important to reiterate that the mean and standard deviation obtained in this manner are different and more meaningful than those attained from arithmetic calculations.

Similar treatments can be used for AR and circularity. Since we define AR in a way that is bounded by 1 and stretches to infinity, a normal distribution is again incorrect to use. Instead, a gamma distribution can be used. Since the values of circularity are bounded by both 0 and 1, we use a beta distribution for that metric. Figure 5 shows fits of the size, AR, and circularity to log-normal, gamma, and beta distributions, respectively, as well as the means and standard deviations that are associated with the fit distributions. The code used to fit these distributions is linked in the SI. Additionally, we have hosted a tool for fitting these distributions to data (https://thelearlab.com/tools/lognormalfitter/). Discussions of all of these distributions and their applicability to the populations are given in the SI.

Table 1 collects the values extracted from fitting. To emphasize the differences between obtaining parameter estimates from fitting and arithmetic calculations, we also present the arithmetic values of the means and standard deviations. It is clear that the arithmetic approach can yield values that differ from those attained by fitting statistical models to the data. This begs the question of which approach is to be preferred.

**Table 1:** Values for Mean and Standard Deviation (SD) for the Maximum Feret Diameter, Aspect Ratio (AR) and Circularity^a^

	low-contrast image		high contrast image	
quantity	value from fit	arithmetic value	value from fit	arithmetic value
maximum Feret mean	2.30 ± 0.026 nm	2.4052	14.22 ± 0.092 nm	16.0039
maximum Feret SD	0.80 ± 0.036 nm	0.7828	0.900 ± 0.095 nm	4.50733
AR mean	1.280 ± 0.075	1.3761	1.129 ± 0.080	1.2194
AR SD	0.03496 ± 0.00264	0.3746	0.00616 ± 0.00056	0.3199
circularity mean	0.6977 ± 0.0154	0.6899	0.8565 ± 0.0584	0.8636
circularity SD	0.1057 ± 0.0150	0.1110	0.0908 ± 0.0564	0.1205

a Presented are the values obtained by fitting distributions to the data (Figure 5) and taking the arithmetic values from the population. Importantly, only the fitting of distributions to the data provides a reliable estimation of the uncertainty in these values (as indicated by the number after the ± symbol). These uncertainties are not present in the arithmetic calculation. More importantly, the value of the standard deviation is not the uncertainty in the mean value.

To begin with, the arithmetic estimate of the mean assumes a symmetric distribution, and the arithmetic estimation of the standard deviation is derived from an assumption of a Gaussian distribution. In practice, these values are almost always interpreted in a manner that assumes a Gaussian distribution. Thus, the more closely the population distribution approximates a Gaussian, the more accurate the arithmetic estimation of the mean and standard deviation will be.

For the case where the population is best described by a log-normal fit, the distribution will approach that of a Gaussian as the value of σ/μ approaches 0. In other words, when the mean is significantly larger than the standard deviation, there will be few values that approach the 0 boundary, so the effects of this boundary are not strongly felt and the resulting distribution can be reasonably approximated as a Gaussian. This fact is illustrated in Figure S8.

Of course, the above reasoning implies that the treatment of the distribution is *always* an approximation. Thus, the estimates will *always* be in error, and the question is how much error is one willing to accept. Again, see Figure S8 and the text that accompanies that figure. We do note that fitting log-normal distributions to these distributions is not significantly challenging. Multiple tutorials exist (https://real-statistics.com/normal-distribution/log-normal-distribution/). Additionally, our group hosts a tool that can be used by anyone to fit a log-normal distribution to data (https://thelearlab.com/tools/lognormalfitter/), and this tool is included in the available data archive linked in the SI. Thus, we generally recommend fitting distributions to the data. As long as the underlying statistical model is chosen correctly, the values obtained from the fitting are physically more meaningful.

Another benefit of fitting distributions to our data is that the results of fitting can be used to obtain uncertainties in the parameter estimations (i.e., the standard error in the parameter values). A misunderstanding that pervades the literature on nanoparticles is the conflation of standard deviation with the uncertainty or “error” in the mean. However, it is simply not the case that these are equivalent. For instance, the analytical expression for the normal distribution has a mean of 0 and a standard deviation of 1. However, the standard deviation of 1 in no way indicates that the mean is uncertain—indeed, it is precisely 0 by definition. Thus, the standard deviation is a measure of the spread of the values but *not* the uncertainty in the mean. Another way to consider this is that super-resolution imaging often leverages the fact that we can know the center of a Gaussian blurred feature with far greater precision than its standard deviation.

In order to obtain meaningful uncertainties for the mean and standard deviation, one can again turn to the fitting of distributions to the data, which can naturally provide uncertainties in the parameters extracted, and these are also reported in the histograms shown in Figure 5.

The utility in having these uncertainties should be immediately obvious when comparing the circularity values of our two samples. A natural question to ask might be whether the two samples have different circularities. Though they both appear to be spheroidal, differences in the circularities could speak to how irregular they are. To answer this question, we can perform a Student’s *t* test on the circularity means using the errors provided from the fit. Doing so provides a *p* value of 0.0086, meaning that the difference in the means is statistically significant. In other words, the smaller particles are meaningfully rougher than the larger particles. Of course, a question remains if this is a result of the relative greater contribution of pixel error (i.e., the fact that pixels are not round) to smaller particles.

It is worth emphasizing the above point: without the values from fitting, the above analysis cannot be done. In the best-case scenario, no statement could be made, as the uncertainty in the mean value is unknown. It is of course possible to estimate an uncertainty using the number of measurements and the standard deviation, but that approach assumes that the underlying distributions are Gaussian—which they clearly are not. Thus, that approach would be incorrect, although the degree to which it is wrong would depend on how strongly the underlying distribution deviates from Gaussian behavior, as discussed above. In the worst-case scenario, one might mistakenly use the arithmetic standard deviation in place of the uncertainty when performing the *t* test. This treatment would lead to the conclusion that the circularities are not different (*p* value of 0.289) when they actually are different. Though in this particular case we are missing significance, it is important to note that one could also experience the opposite case: concluding that two values are significantly different when they actually are not. Clearly, proper treatment and reporting of measured properties is critical for proper scientific analysis.

## 4 Comparison of Different Approaches

With a discussion of reporting out of the way, we can return to a comparison of the different methods available for processing particle images. Because size dominates the discussion of particles in the literature, we will perform this comparison using size measurements. Additionally, we perform this discussion using the high-contrast image shown in Figure 1B. We use this image because it is the one for which manual processing and thresholding will work best; therefore, any advantage seen for Weka segmentation will only improve for lower-contrast images. Additionally, we note that there are not 200 particles present in this image. Thus, the fitting discussed here is not meant to be an accurate representation of the particles but to allow easy comparison between techniques.

### 4.1 Weka Segmentation versus Thresholding for Particle Identification

Both thresholding (Figure 1C,D) and Weka segmentation (Figure 1E,F) allow for automated particle identification. However, Weka segmentation requires more time to implement with respect to both the initial training and its application to images. Above, we demonstrated that thresholding produces a messy result for low-contrast images, so Weka is clearly preferred for such images. However, it is worth considering whether thresholding is a reasonable alternative for images with high contrast. To this end, we applied both Weka segmentation and thresholding to the image in Figure 1B. From the outlines obtained, we created the histograms shown in Figure 6. These histograms represent the sizes of the particles attained by the maximum Feret diameter and derived from the area (assuming a circular shape) for both Weka-segmented and thresholded images. These histograms also contain fits of a log-normal distribution to the data, and the extracted means (called “geometric means”) and standard deviations are shown with their respective uncertainties. We also calculate the arithmetic means and standard deviations. All of these values are shown in the bar charts for the mean (Figure 6E) and standard deviation (Figure 6F). In these bar charts, the error bars represent the standard errors.

**Figure 6:**
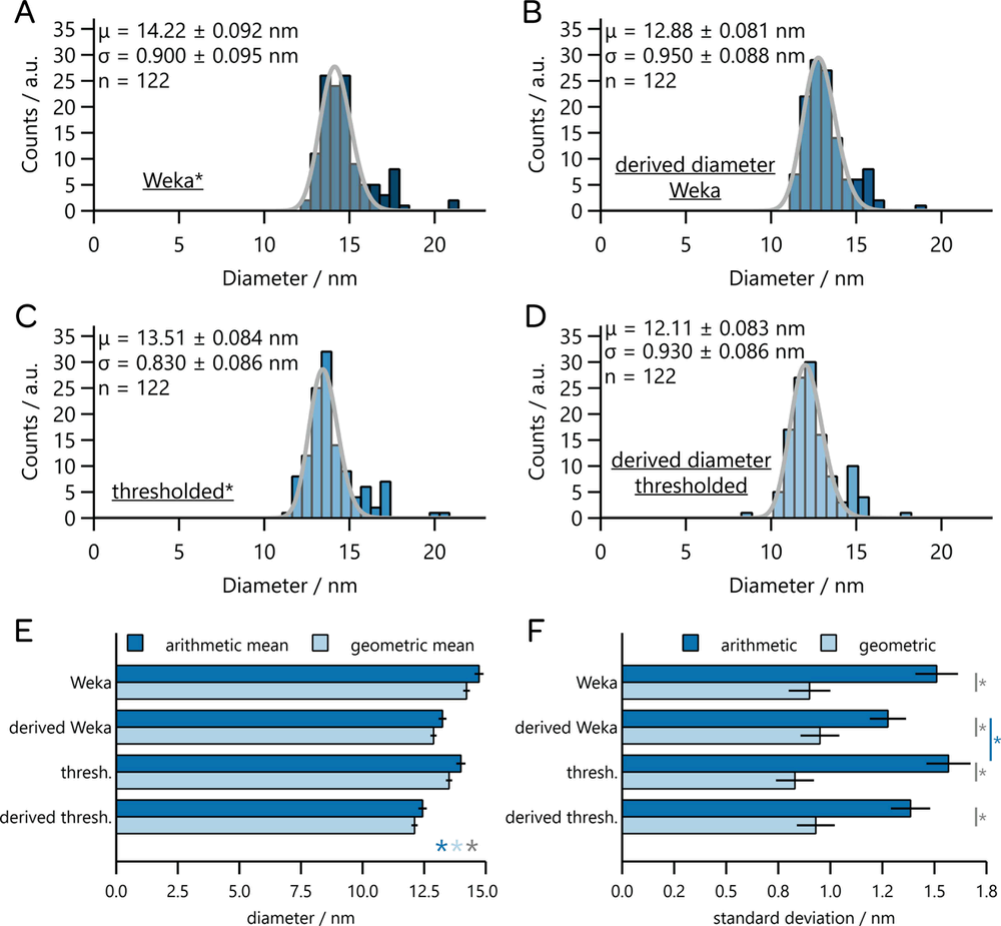
Histograms associated with diameter measurements of particles in the high-contrast image obtained through different methodologies. Each dataset was fit to a log-normal distribution. The methodologies used Weka segmentation* (A), derived diameter from Weka area (B), thresholding of the raw image*, (C), and derived diameter from the thresholded particle area (D). (E, F) Statistical comparison of the arithmetic and geometric means (E) and standard deviations (F) at α = 0.05 using Welch’s *t* test. *Indicates that the dataset was manually edited to remove aggregate particles and single-pixel measurements.

The most important result of this work is that every pair of Weka-segmented and thresholded images produce means that are statistically significantly different based on a Welch’s *t* test, with a threshold of significance (α) of 0.05. The reason this is an important result is that it suggests that one should not use different approaches to image processing on images that one wishes to compare, as this will very likely produce results that are different—no matter if a real difference exists. However, it is also interesting to note that, for both parameters, the geometric (i.e., obtained from fitting) and arithmetic parameter values are statistically significantly different from each other—suggesting that these particular distributions are not close enough to Gaussian to use arithmetic calculations of these parameters. Finally, we note that in all cases the geometric values are smaller than the arithmetic values, again showing the systematic errors that can arise from using arithmetic calculations of these values.

Given these results, we suggest that Weka segmentation is generally preferred to thresholding. The reason is the same as the first point in the above paragraph: one should not change approaches between images. Given that thresholding will be a problem for low-contrast images, using Weka segmentation for even high-contrast images will allow for a comparison between both low- and high-contrast images. It is also fortunate that the time needed for training and application of Weka segmentation is shortest for high-contrast images, somewhat ameliorating the disadvantage of Weka segmentation for these images.

### 4.2 Comparing Results from Weka Segmentation and Manual Sizing

Another proposed benefit of using Weka segmentation is that it could provide greater repeatability and reproducibility—especially compared with manual processing. Here we use repeatability to indicate the ability of a single researcher to reproduce their analysis on an image and reproducibility to indicate the ability of different researchers to reach the same result.

Regarding repeatability, once a Weka model is trained, it can be applied to the same image with effectively no change in the results. Thus, repeatability is very high at the point of application. There is a question of repeatability in terms of training, but in our experience, changes are so small as to produce no statistically significant difference in the outcome.

To test reproducibility, we had five researchers from our group analyze the high-contrast image (Figure 1B) both using Weka segmentation and manually. These researchers spanned experience levels ranging from one of the authors of this paper to a first-year undergraduate student who had joined the group 6 weeks prior.

Each researcher was provided instruction on how to size. For manual sizing, a demonstration of how to use the FIJI line tool was provided. The researchers were then given verbal explanations to measure what they perceived as the longest chord of each particle and to exclude aggregate particles. Aggregate particles were determined at the discretion of each researcher. Similarly, a demonstration was given for how to use the Trainable Weka Segmentation, and they were given the guide provided in the SI. Settings for Weka segmentation were maintained at the default values with a small addition of checking the Balance classes option. Once the results were obtained, they were allowed to remove aggregate and single-pixel measurements from their datasets. For the analysis following Weka segmentation, all researchers adjusted the Set Measurements options to include Area, Min & max gray value, Shape descriptors, Mean gray value, Centroid, and Feret’s diameter and to increase the Decimal places (0–9) to 5 (originally 3).

For both manual and Weka-segmented measurements, fitting and statistical analysis of the data were performed by the authors of this paper using the tool hosted by our group (https://thelearlab.com/tools/lognormalfitter/). The results of fitting log-normal distributions, the parameters extracted from these fits, and the numbers of particles measured (*n*) are shown in Figure 7. The left column (Figure 7A–E) contains the results for manual measurements, while the right column (Figure 7F–J) contains the results from Weka segmentation.

**Figure 7:**
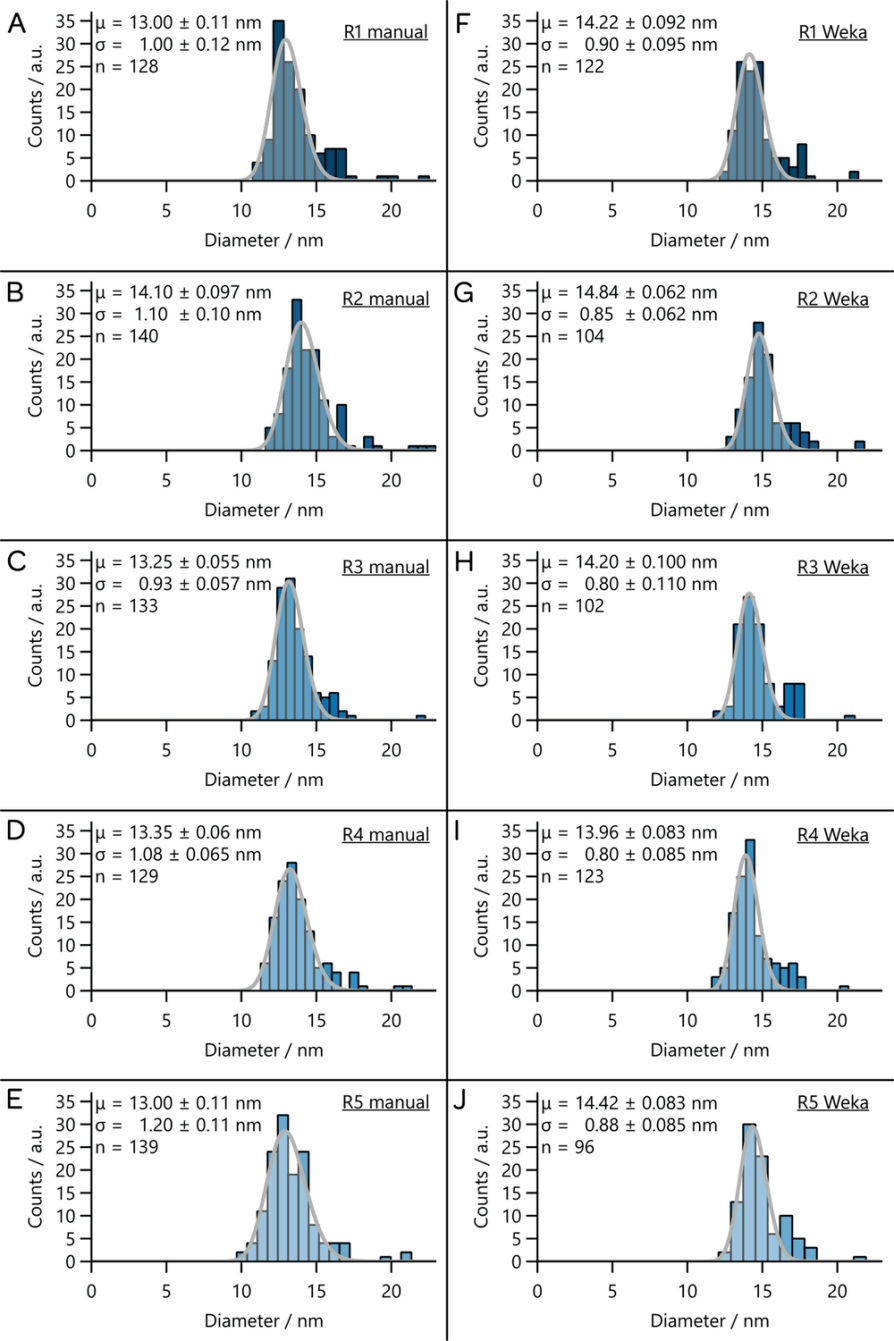
Histograms associated with diameter measurements of particles in the high-contrast image through manual (A–E) and Weka segmentation (F–J) performed by different researchers. Each dataset was fit to a log-normal distribution. The left column corresponds to manual measurements, while the right column is for Weka segmentation followed by manual removal of aggregates by the respective researcher.

There are several aspects of these results that are worth commenting on:

- *For each researcher, the number of particles (n) identified in the image is always smaller for Weka segmentation than for manual measurements.* Moreover, if we compare the means of particles counted (⟨*n*⟩), then the means are statistically different (*p* = 0.008). This suggests that either Weka segmentation is missing particles that researchers manually included or that the exclusion of particles after Weka segmentation is more rigorous than the exclusion being done for the manually measured particles. We think that the latter is more likely, though we did not examine this in detail.
- *For each researcher, the mean size (μ) obtained from the manual measurements is always smaller than the mean size obtained from Weka segmentation.* However, this difference is not always significant at a *p* value of 0.05. Additionally, if we compare the mean of means (⟨μ⟩) for manual and Weka treatments, we do find a statistically significant difference (*p* = 0.00088). This trend may be due to bias in determining bounds of the nanoparticles, with a more conservative approach taken by humans to ensure that background is not included.
- *For each researcher, the standard deviation of the distribution (σ) attained from Weka segmentation is always smaller than the standard deviation attained from manual sizing.* Similar to the mean, these differences are not always significant. Again, when we compare the mean value of the standard deviation (⟨σ⟩) for each group, we find a statistically meaningful result (*p* = 0.0012), meaning that the Weka segmentation also produces a meaningfully more narrow set of distributions compared to manual sizing. For this result, we suggest that researchers are again a bit worse at providing consistent identification of the edges of the particles, leading to a broader distribution of sizes identified. However, this could be due to inclusion of more outliers in the manual distribution, consistent with the results from the first point.
- *The standard deviation of particles counted (σ_n_) is larger for Weka segmentation than for manual processing.* We note that these standard deviations are not statistically significantly different (*p* = 0.151). We suspect that the larger distribution for the Weka segmentation is a result of the quality of training. Since the processing used rejects large particles, we believe that the smaller numbers of particles counted for R3 and R5 are likely due to training that did not provide good segregation of clusters, thereby leading to rejection of these clusters—and a fairly large number of particles. We do note that the particle sizes obtained are not strongly affected by the smaller values, suggesting that the particle exclusion was not systematic—in other words, the clusters are formed of randomly drawn particles from the sample.
- *The standard deviation of means (*σ*_μ_) and the standard deviation of standard deviations (*σ*_*σ*_) are smaller for the Weka-based measurements than for the manual measurements.* However, neither is statistically significantly different (*p* = 0.0504 and *p* = 0.997, respectively). Here we wish to emphasize the small size of this study (five researchers). It could very well be that looking at a larger set of researchers (e.g., across a field of study) would result in significant differences, though conducting such a large study lay far beyond the scope of this tutorial. Nevertheless, it can at least be said that the higher-throughput Weka segmentation can be used without increased variance in measurements between researchers and could potentially decrease this variance at larger scales. It is also interesting to note that, even though there was a larger σ_*n*_ for Weka than for manual, the σ_μ_ and σ_σ_ were not larger. This again suggests that this was due to excluding aggregates comprising representative particles rather than systematic bias in the size of the particles that were excluded.

In total, we found that manual processing and Weka-segmentation-based processing produce results that are statistically different from one another. Similarly to the discussion above regarding thresholding, this suggests that the best practice when attempting to compare two groups of particles is to use the same approach to image processing.

Additionally, we note that all of the above discussion is based on the analysis of a *single* image. In reality, particle characterization would likely be done on a number of images, both in order to ensure that a larger number of particles can be counted and to ensure that characterization is not dependent on a single subpopulation. The latter is a critical consideration, as particles can segregate into subpopulations on a TEM grid. We anticipate that the relative advantage of Weka segmentation will only increase with the number of images processed. Certainly, the time for processing will be decreased, but this also means that a larger number of particles can be counted, which will produce better estimations of the mean (Figure S9). Thus, in general, we believe that Weka segmentation offers several advantages and will likely represent a benefit for most research groups that are currently sizing particles manually. However, there are a few caveats that are worth considering, to which we now turn.

## 5 Caveats Regarding Weka Segmentation

Like many scientific tools, Weka segmentation is used to aid researchers in achieving faster, less biased, and/or more reproducible analysis. However, it is important to note the weaknesses of such a machine learning tool. While there may be many caveats, we will limit our discussion to the two most pressing issues: the particle identification issue and software constraints. Of course, segmentation does nothing to mitigate errors in the TEM acquisition, so one should also ensure proper sampling of the TEM mesh, the use of proper magnification, etc. Though discussion of the proper sampling lies beyond the scope of this tutorial, we note that it is still critical to consider.

Automatic particle identification is the main utility that we illustrate in this tutorial. However, indiscriminate segmentation of overlapping particles is more than apparent, as can be seen in Figure 8. In cases where the boundaries of the particle are close in proximity (1–2 pixel boundary), training the classifier and modifying settings may allow differentiation of the two. In the more extreme cases of overlap, the easiest way to alleviate erroneous measurements of overlapping particles is when analyzing the thresholded image in FIJI. A dialogue box is opened once Analyze Particles is selected. In this box, there is an option labeled Size (nm^∧^2). Assuming that the overlapping particles are much larger than the singular particles, an upper size limit can be designated. This will ensure that when FIJI analyzes the image it will exclude any measurements larger than the upper bound. However, due to the nature of nanoparticles, nanoparticle size often has slightly heterogeneous distributions. Thus, limiting size should only be performed if necessary and in such a way that it does not remove valuable and real measurements that impact the distribution. A more judicious approach is through the manual removal of such data points. Each measurement will be given an outline and a number inside the outline if Show: Outlines is selected when analyzing the image. The number aligns with the measurement number in the result output. The measurement corresponding to the overlapping particles can then be easily identified and manually removed. This was the approach used in the comparisons described above. While this method does require a greater time investment, it ensures more accurate processing of the data.

**Figure 8:**
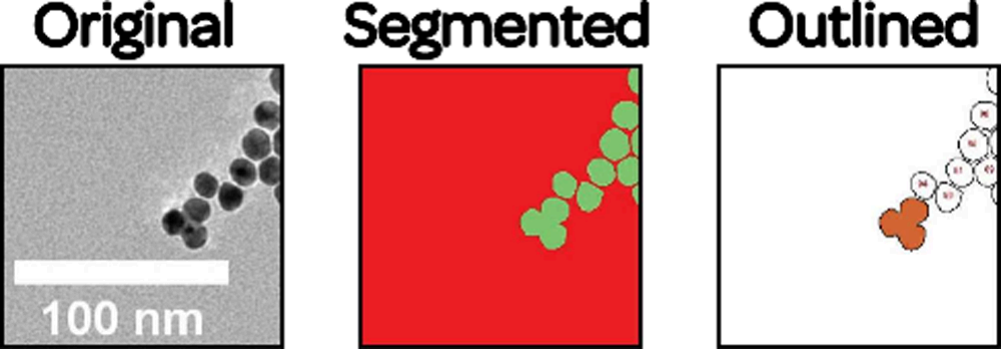
Comparison of aggregate particles maintained throughout the segmentation process.

Beyond particle identification, the software also has constraints. These constraints are particularly evident when working on large (>16 MB) or very low contrast images. On midrange computers (16 GB RAM), Weka can function decently if the image size is at or below 16 MB (2048 × 2048 pixels) and the memory allocated to FIJI has been increased. The segmentation process can be completed on a high-quality image in seconds to minutes. However, the same image but at a larger size, 64 MB (4096 × 4096 pixels), may encounter memory errors. Similar errors may occur with incredibly low contrast images (16 MB). On the low-contrast image, the error may be alleviated through additional sampling of each respective class. Additionally, sampling is a balance. While more sampling provides a larger dataset for the tool to train and learn on, it comes at a longer training time. Smaller sampling often leads to less adequate training but occurs much faster. However, more data may need to be added to the classes, and further training may be required. This balance was specifically illustrated with researcher R5 (see above), in which the first training took over an hour due to high sampling for both classes.

## 6 Conclusions

In this tutorial, we have outlined some of the challenges associated with manually analyzing TEM images of nanoparticles as well as a solution to these challenges using the free Weka segmentation plugin of the popular image analysis software FIJI. We have demonstrated how segmentation using this plugin provides more robust and consistent identification of particles and also enables fast determination of measurements of size and morphology. We have also suggested that Feret diameter can be used as a robust measurement of size, fitting of an ellipse to particle shapes can be used as a robust measurement of aspect ratio, and circularity is another metric that can be used to discuss the morphology of particles. All three of these can be determined within FIJI following Weka segmentation. Finally, we have discussed some best practices for reporting these measurements, with a specific focus on understanding how to obtain meaningful values for the mean and standard deviation of the measurements as well as the dangers associated with conflating the standard deviation with the uncertainty in the mean value. In total, we hope that this tutorial will enable researchers to increase the throughput of their experiments while simultaneously increasing the accuracy and power of their measurements.

## Supplementary Material



## Data Availability

An archive of all data and Python codes can be obtained upon request and found at: https://doi.org/10.26208/6PEX-RH37

## Notes

Step-by-step description of how to use the Weka segmentation in FIJI, procedures for generation of histograms and distribution fitting, description of log-normal versus Gaussian distributions, and discussion of dependence on the number of samples (PDF)
