# Supplementary material for: Practical Guide to Automated TEM Image Analysis for Increased Accuracy and Precision in the Measurement of Particle Size and Morphology
Source: ACS Nanosci Au. 2025 Apr 17;5(3):117–27. doi: 10.1021/acsnanoscienceau.4c00076 (PMC12186846; doi:10.1021/acsnanoscienceau.4c00076)
Supplement: Supplementary file 1 [file ng4c00076_si_001.pdf]

Supporting Information: Practical Guide to Automated TEM Image Analysis for Increased Accuracy and Precision in the Measurement of Particle Size and Morphology

Kristen Aviles and Benjamin J. Lear*

Department of Chemistry, The Pennsylvania State University, University Park, 16802 USA

E-mail: bul14@psu.edu

Contents

1 Data archive **S-3**

2 Step-by-step Guide **S-3**

3 Generation of a histogram **S-11**

4 Distribution Fitting **S-12**

4.1 Nanoparticle Size - Lognormal Distribution S-13

4.2 Nanoparticle AR - Gamma Fit S-14

4.3 Nanoparticle Circularity - Beta Fit S-15

4.4 Example data S-16

5 Gaussian versus lognormal distributions **S-17**

6 Dependence on number of samples **S-20**

1 Data archive

All data used in this manuscript, Python code used to process the data, images used in the manuscript, and Veusz files can be obtained at the Penn State data commons archive at: https://doi.org/10.26208/6PEX-RH37

2 Step-by-step Guide

Below, we provide a step-by-step guide to using the trainable WEKA segmentation in FIJI. The data used to produce the images shown in the main manuscript is availible at https://doi.org/10.26208/6PEX-RH37 and can be used if you wish to produce the same result. Additionally, Figure S5 provides a flow chart for this process and can be used in conjunction with this step-by-step description to understand the use of this FIJI plugin.

Classifying the image:

1. Open the microscopy image in FIJI. Any 2D image can be used. It is suggested to use sufficiently lossless file types such as .tiff or .ser.

2. Select the Trainable Weka Segmentation plugin. To navigate to the plugin use the FIJI tool bar. It is located under `plugins > segmentation > Trainable Weka Segmentation`. This will open the selected image from FIJI into TWS.

3. Adjust the settings for your segmentation needs. Default settings are adequate for a two-class image (i.e., nanoparticles and mesh). The settings are the last button on the left side panel as illustrated in Figure S1. Here you can modify blurs, classifiers, features, and class names.

    - More classes can be added using the `Create a New Class` option in the left panel. Once a name is inputted to the class, select `OK`. This will add the new class on the right panel.

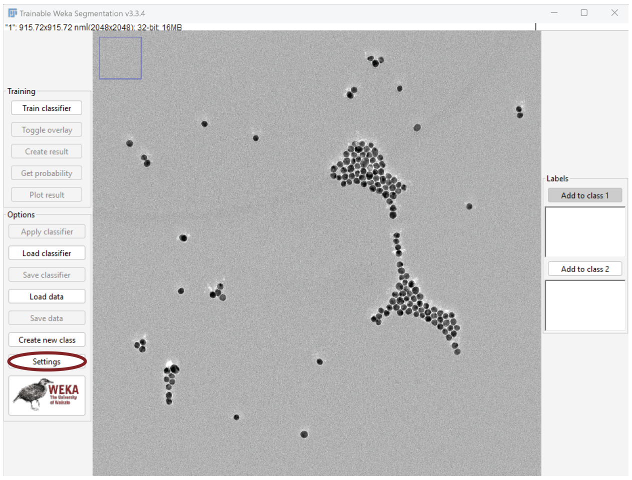

Figure S1: Settings location in Weka are circled in red.

4. Sample the image and add the selected area to the corresponding class. To sample, select one of the shape tools on the FIJI toolbar. For more control on selections use the `Freehand selections` tool as seen in Figure S2. Once selected use the `Add to Class` button(s) on the right.

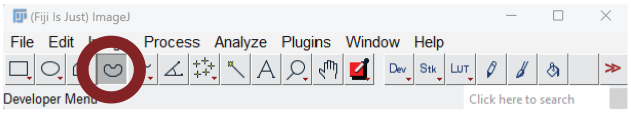

Figure S2: `Freehand selection` tool location in FIJI is circled in red.

5. Train the Classifier. Select the `Train Classifier` button at the top of the left panel.

   - Classifier training time will be variable dependent on the image size, memory allocated to FIJI (memory available on the device), number of classes, training features selected, and classifier type. To change the memory FIJI is allowed to use navigate to `Edit` in the FIJI toolbar. Under `Options` select `Memory & Threads`.

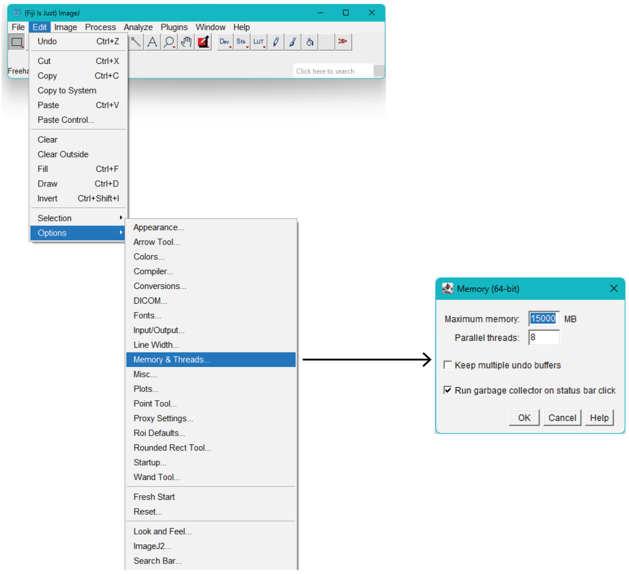

Figure S3: To change the memory that FIJI is allocated to use navigate in the FIJI Tool Bar to `edit > options > memory & threads`. This will open a new dialog box with an option to change the maximum memory.

- A red and green overlay will be displayed in the Weka Plugin once training is complete. This segmented image can be toggled using the `Toggle Overlay` button.
- If the segmented image does not appear adequately segmented, repeat steps 4 and 5.
- The colors can be modified in Weka by right-clicking the `Add to Class` buttons. This will open a new window with a color selection tool.

6. Create Result. Select the `Create Result` button near the bottom of the left panel. This will open the red and green segmented image into FIJI.

7. Optional: Apply the classifier to other images. Otherwise, skip to Analysis of the Image section.

   - If continuing to train in the *same session* (i.e., Weka is open and the original image is still active), select the `Apply Classifier` button on the left panel. Then select the image. (Caution: While you can train a classifier on a .ser file, you cannot

apply a classifier to a .ser file. However, you may convert .ser files to .tiff to apply classifiers.) A pop-up will appear asking "Create probability maps instead of segmentation?" Select `No`. Once the selected image is segmented it will open in FIJI as the red and green image and as the original.

- The classifier can also be saved for future use. To reload a saved classifier, an image must be opened in the Weka plugin. Select `Load Classifier` and then choose the saved model. Once loaded, the classifier can be applied to other images by selecting `Apply Classifier` and following the step above. If Train Classifier is selected an error will occur because there are no samples added to the classes. `Apply Classifier` must be used to segment new images with a trained classifier.
- If the image types (8-bit, 16-bit, RGB Color, etc.) are different between the image the classifier was trained on and the image the classifier will be applied to a "`Could not apply Classifier!`" error message may appear. To circumnavigate this issue, the type can be modified in FIJI by opening the image intended for classification, navigating to `Image` then `Type`, then selecting the corresponding type as illustrated in Figure S4.

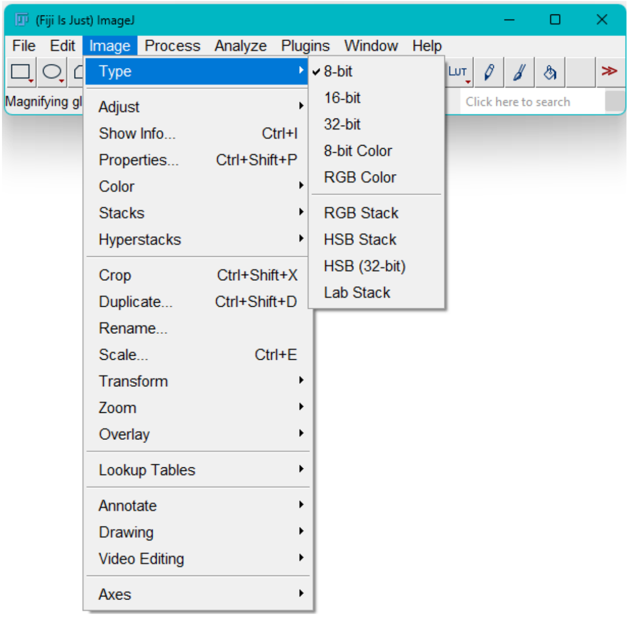

Figure S4: To change the image type navigate in the FIJI Tool Bar to `image > type`. This will show multiple options. Select the one that matches the image type of the image the classifier was trained on.

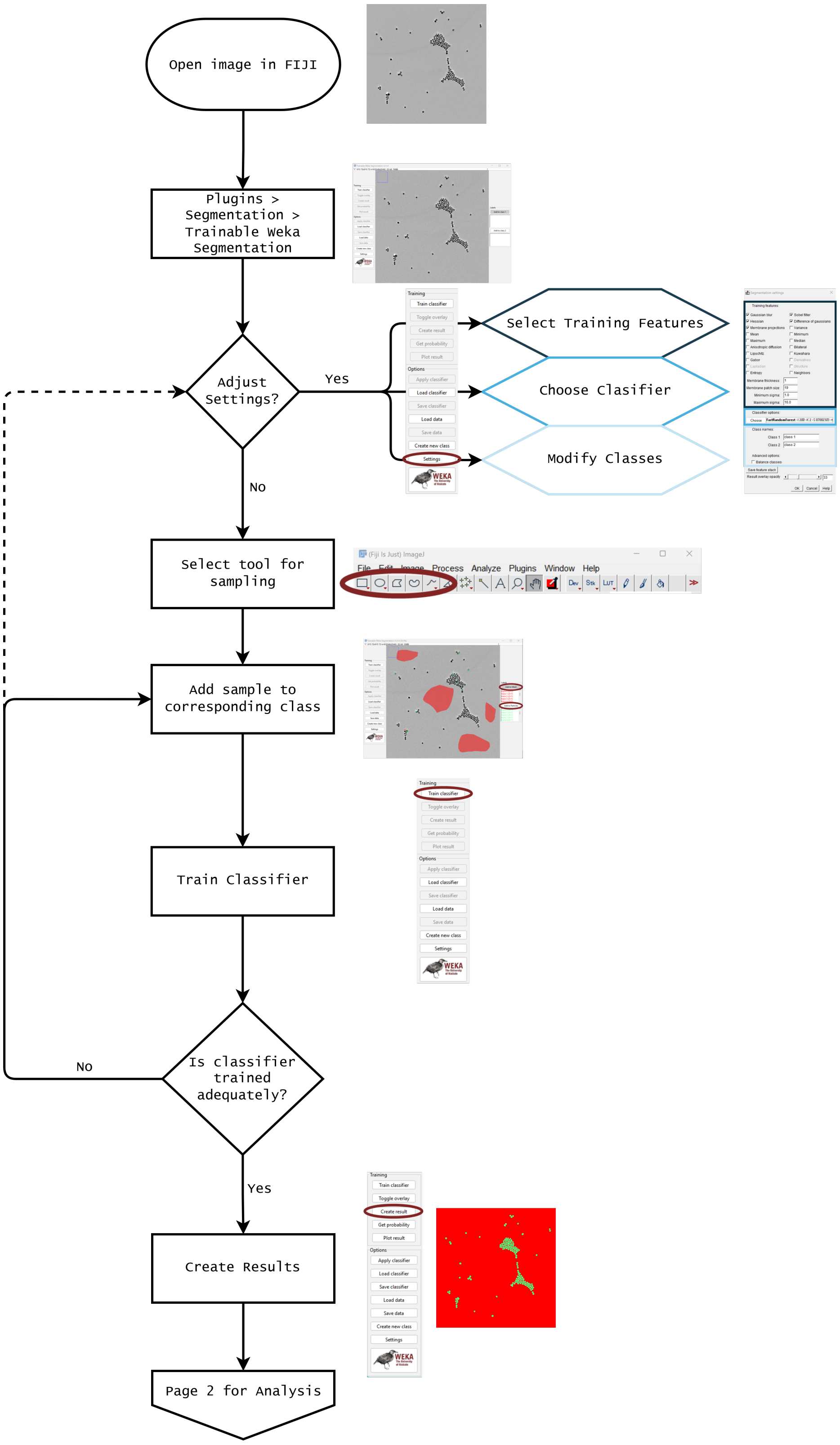

Figure S5: A flowchart illustrating the steps highlighted in Classifying the image guide. Key buttons/tools are circled in red.

Analysis of the image:

1. Binary threshold the image. There are several approaches to do this. The easiest way is by first changing the image to 8-bit. To do so, locate Image in the FIJI toolbar. Select `Image`, then `Type`, then `8-bit`, refer to Figure S4. This will create a grey-scale image. To then obtain a binary image navigate to `Image, Adjust, Threshold` or `control + shift + T`, select `Apply`, and close the thresholding window. Ensure all options are consistent with Figure S6.

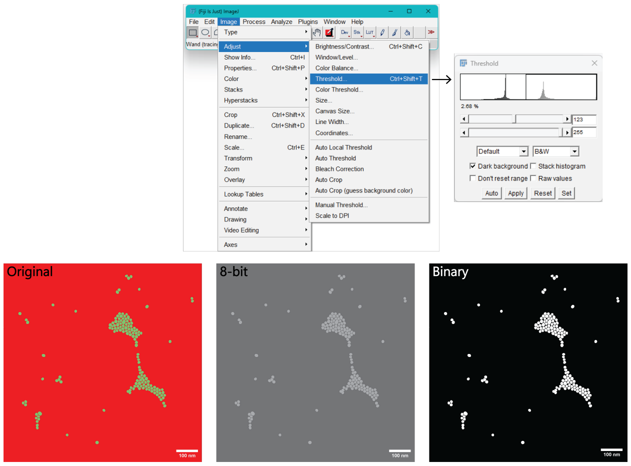

Figure S6: To modify the segmented image to a binary image, first adjust the image to 8-bit then navigate to `image > adjust > threshold` or `control + shift + T`. Once thresholded the image can features can then be analyzed with FIJI.

2. Analyze the particles. Select `Analyze > Analyze Particles` in FIJIs toolbar.

   - Measurements can be selected beforehand by navigating to `Analyze > Set Measurements`.
   - In the Analyze Particles pop-up, there will be several options including Size limits, Circularity limits, and Show. We recommended to select `Outlines` from the Show drop down menu and to check the boxes for `Display results, Clear results, Exclude on edges, and Overlay`.
   - Two windows will open: one with tabulated measurement values labeled `Results` and one with the corresponding outlined particles. The values in the `Results` window can be saved as a .csv file by selecting `File > Save As` or `control + S`.

- Two errors may be encountered during this step. The first will result in an error dialog box opening stating, "`No particles were detected...`". While the second may produce an outputted image that does not have the outlines of the particles. In the latter case, measurements are still made but rather on artifacts of the background. For either case, this may be due to a thresholding issue. To resolve these issues, navigate to `image > adjust > threshold`. In the thresholding window, select or deselect `Dark background` then select `Apply` and retry the `Analyze Particles` function.

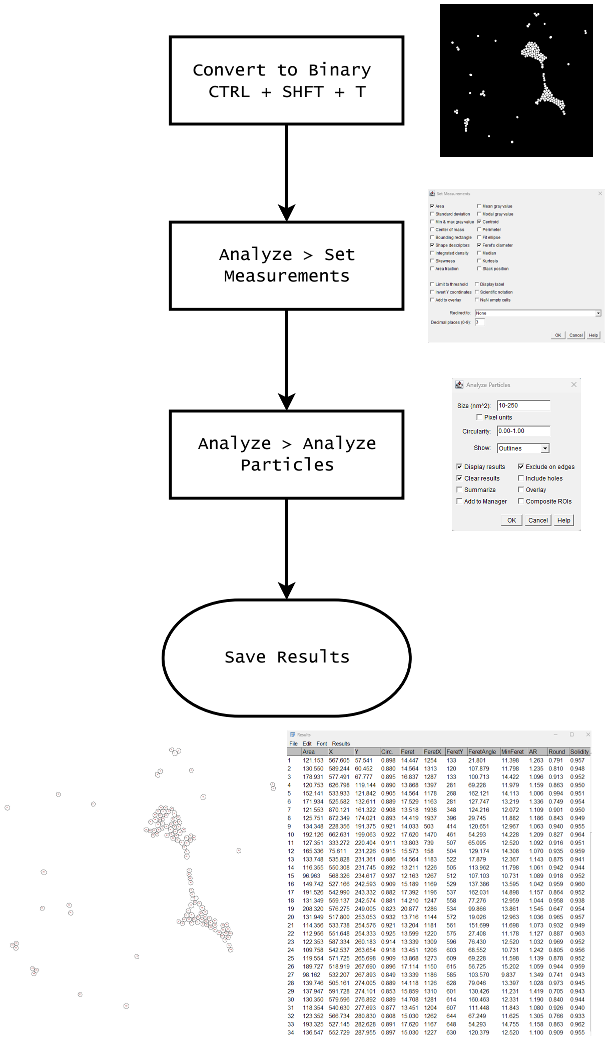

Figure S7: A flowchart illustrating the steps highlighted in analysis of the image. The bottom image displays the given output and the calculated results when outlines are selected.

3 Generation of a histogram

The histograms shown in the main manuscript and in this supporting information were constructed using either Plotly within Python, or using Veusz (a free and open source plotting program with a graphical user interface) which can be obtained at: https://veusz.github.io/ Exemplar Veusz files for the plots shown in the main manuscript can be found at https://doi.org/10.26208/6[illegible] RH37 In both cases, they were constructed as bar charts in the following manner.

- *We calculated size of each bins to be used.* This is done in several steps. First the interquartile range (IQR) of the data is calculated. This is the middle 50% of measurements and is most readily done by sorting the values in order from largest to smallest and then finding the value at position $0.25 \cdot n$ and $0.75 \cdot n$, where n is the number of particles counted. The difference between these two values is the IQR. With the IRQ in hand, one can find the size of the bins (w) using the Feedman-Diaconis rule:

$$w = 2(\text{IQR})(n)^{1/3} \tag{1}$$

  where n is the number of particles counted.

- *The bin widths are then used to identify the edges of the bins* using:

$$\text{edges} = \{a + k \cdot w \mid k \in Z, a + k \cdot w < b + w\} \tag{2}$$

  where a is the smallest measured particle, b is largest measured particle, k is the integer index, and Z is the set of integers.

  We note that we are using the Freedman-Diaconis rule, though there are other guidelines that are also widely used.

- *The number of values occurring between each lower and upper bin edge is counted up.* This yields the 'counts' for each bin. We are not almost ready to plot. Since we are

going to plot this as a bar chart, two steps remain.

- *The location of the bars (the center of each bin) is found* using:

$$\text{centers} = \frac{\text{edges}_j + \text{edges}_{j+1}}{2}, \quad \text{for } j = 1, 2, \ldots, n-1 \tag{3}$$

where edges are the boundaries of the bins, j is the bin index, and n is the number of bin edges.

- *Construct a bar chart* where the length of the bars are the counts, and the bars are positioned at the bin centers. The bar chart is also adjusted so that the bars are wide enough to touch one another.

This completes the formation of the histogram. There are two advantages to constructing a histogram in this manner. First, it is easy to format and label and is widely availible within many plotting packages. Second, it naturally produces values for the bin centers and the bin counts, which are used in the fitting of distributions to the histogram.

4 Distribution Fitting

No matter the distribution chosen, the same basic strategy is employed. First, the model distribution is chosen. Second, initial guesses at the distribution parameters are made. Third, these guesses are refined in order to minimize the sum of squares between the calculated distribution and experimental histogram. For this manuscript, fitting was done using the `lmfit` library of Python. The code used is availible at the archive at https://doi.org/10.26208/6PEX-RH37

4.1 Nanoparticle Size - Lognormal Distribution

The diameter of nanoparticles is a positive continuous property. The lognormal probability distribution is used to fit this property:

$$f(x;\mu,\sigma) = \frac{1}{x\sigma\sqrt{2\pi}} \exp\left(-\frac{(\ln x - \mu)^2}{2\sigma^2}\right),\ x \in (0,\infty) \tag{4}$$

Where μ is the mean, σ is the standard deviation. To fit the distribution, we first estimate guesses for the mean and standard deviation. The mean initial guess is calculated by using the arithmetic mean formula:

$$\mu_{\text{initial}} = \bar{x} = \frac{1}{N}\sqrt{\sum_{i=1}^{N} x_i} \tag{5}$$

Where N is the number of data points and x_i is the i-th data point. The standard deviation initial guess is calculated by:

$$\sigma_{\text{initial}} = \sqrt{\frac{1}{\mathrm{N}}\sum_{i=1}^{N}(x_i - \bar{x})^2} \tag{6}$$

An in-house python script then executes fitting using the Python module lmfit. The result of the fit includes the estimations of the mean and standard deviation, as well as the estimated standard errors for these values. These values are in log space, but can be converted to real space. The real space mean is calculated by:

$$\mu_{\text{real space}} = \exp\left(\mu + \frac{\sigma^2}{2}\right) \tag{7}$$

The standard error of the real space mean is calculated by:

$$\text{SEM} = \sqrt{\left(\exp\left(\mu + \frac{\sigma^2}{2}\right)\Delta_\mu\right)^2 + \left(\exp\left(\mu + \frac{\sigma^2}{2}\right)\sigma\Delta_\sigma\right)^2} \tag{8}$$

The real space standard deviation is calculated by:

$$\sigma_{\text{real space}} = \sqrt{(\exp(\sigma^2) - 1)\exp\left(2\mu + \sigma^2\right)} \tag{9}$$

Lastly, the real space standard error of standard deviation is calculated by:

$$\text{SESD} = 0.5\frac{\sqrt{2\left(\exp(\sigma^2) - 1\right)\exp\left(2\mu + \sigma^2\right)\Delta_\mu)^2 + \left(\exp\left(2\mu + \sigma^2\right)\left(2\exp\left(\sigma^2\right)\sigma + \exp\left(\sigma^2\right) - 1\right)\Delta_\sigma\right)^2}}{\sigma_{\text{real space}}} \tag{10}$$

4.2 Nanoparticle AR - Gamma Fit

The aspect ratio of nanoparticles is a positive continuous property. The gamma probability distribution is used to fit this property:

$$f(x; k, \theta) = \frac{(x)^{k-1}\exp(-\frac{x}{\theta})}{\Gamma(k)\theta^k},\ x \in (0, \infty) \tag{11}$$

where k is the shape parameter, θ is the scale parameter, and $\Gamma(k)$ is the Gamma function, where $\Gamma(k) = (k-1)!$. The Gamma distribution is bounded on the low end at 0, but this can be shifted by substituting $(x - b)$ for x, where b is the new desired lower bound. Fitting this equation to data gives estimates of the shape and scale parameters, as well as estimates of the standard errors associated with these parameters. Using these values, the mean is given by:

$$\mu = k \cdot \theta \tag{12}$$

The standard deviation is given by:

$$\sigma = \theta\sqrt{k} \tag{13}$$

The standard errors associated with the mean and standard deviation are arrived at using standard error propagation methods.

4.3 Nanoparticle Circularity - Beta Fit

Circularity is a continuous property defined by two distinct bounds, (0,1). The beta probability distribution function fits such constraints and is defined by:

$$f(x;\alpha,\beta) = \frac{x^{\alpha-1}(1-x)^{\beta-1}}{B(\alpha,\beta)},\ x \in (0,1) \tag{14}$$

where α and β are called shape parameters. $B(\alpha,\beta)$ is defined by:

$$B(\alpha,\beta) = \frac{\Gamma(\alpha)\Gamma(\beta)}{\Gamma(\alpha+\beta)} \tag{15}$$

Fitting the beta distribution to data gives estimates of these shape parameters, as well as the standard errors associated with them. Using these values, the mean is given by:

$$\mu = \frac{\alpha}{\alpha+\beta} \tag{16}$$

The standard deviation is given by:

$$\sigma = \sqrt{\frac{\alpha \cdot \beta}{(\alpha+\beta)^2(\alpha+\beta+1)}} \tag{17}$$

The standard error of the mean associated with the the mean and standard deviation can be obtained using standard error propagation methods.

4.4 Example data

Below is the TEM image used for the Weka instructions provided above. The parent .tiff file can be found in the associated archive file.

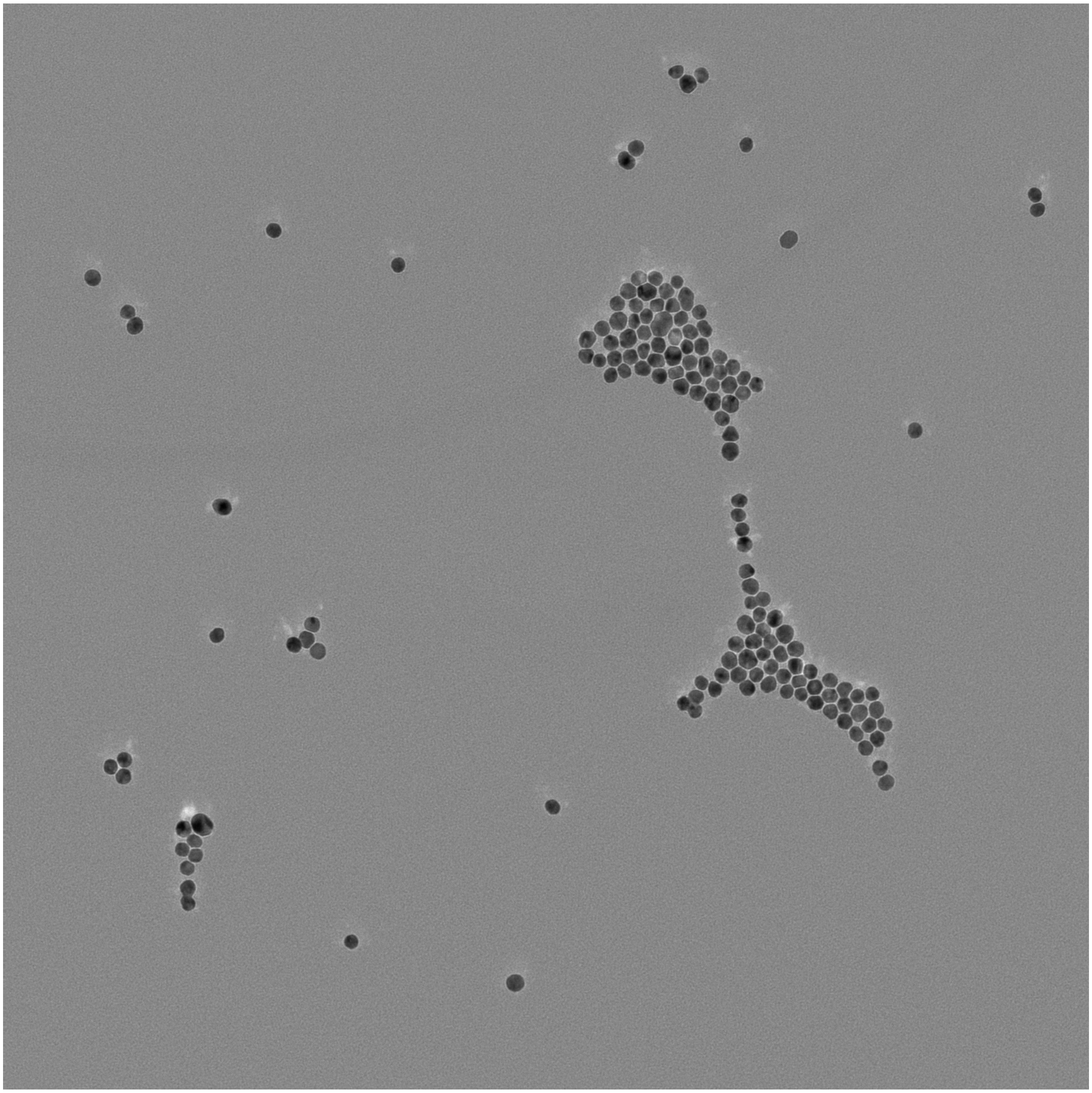

5 Gaussian versus lognormal distributions

One question that can arise when thinking about fitting extracting estimates of means from distributions, is what underlying distribution to use. When thinking about particle size, the choice is often between treating the distribution as Gaussian or lognormal. The desire to treat a distribution as Gaussian stems from the fact that it is that calculation of mean and standard deviation is relatively straightforward. They can be done using simple arithmetic approaches. Additionally, there are ways to estimate the uncertainty in these estimates that are also based on solving simple equations. However, it is also true that no size distribution can be strictly Gaussian, since particle sizes cannot be negative, but all Gaussian distributions have some probability of negative values. At the same time, for certain combinations of mean and standard deviation, the Gaussian distribution may be a sufficiently accurate approximation—especially considering noise and measurement limitations. Thus, the question is how close the Gaussian approximates the lognormal distribution. In other words, the question is how much systematic error a researcher is willing to accept.

To illustrate this point, Figure S8 shows difference between Gaussian (blue) and lognormal (red) distribution for various means (μ) and standard deviations σ. In this figure, the left column is for distributions with a mean of 1, while the right column is for distributions with a mean of 100. Each row in this figure shares a common relative standard deviation ($\frac{\sigma}{\mu}$). Additionally, the dashed vertical red line gives the value of mean that would be attained if the lognormal mean was estimated using the arithmetic mean and the relative error this is from the true mean ($|\frac{\mu_{arithemetic}-\mu_{true}}{\mu_{true}}|$) is also given.

In examining this figure, a few things should be clear:

- As the lognormal distribution becomes narrower, the distribution becomes better approximated by a Gaussian.
- The value of the mean does not not determine the relative error in mean estimation. Instead, it is entirely due to the relative standard deviation ($\frac{\sigma}{\mu}$). This reflects the fact that a lognormal cannot have x-values less than 0, and so when the distribution is wide

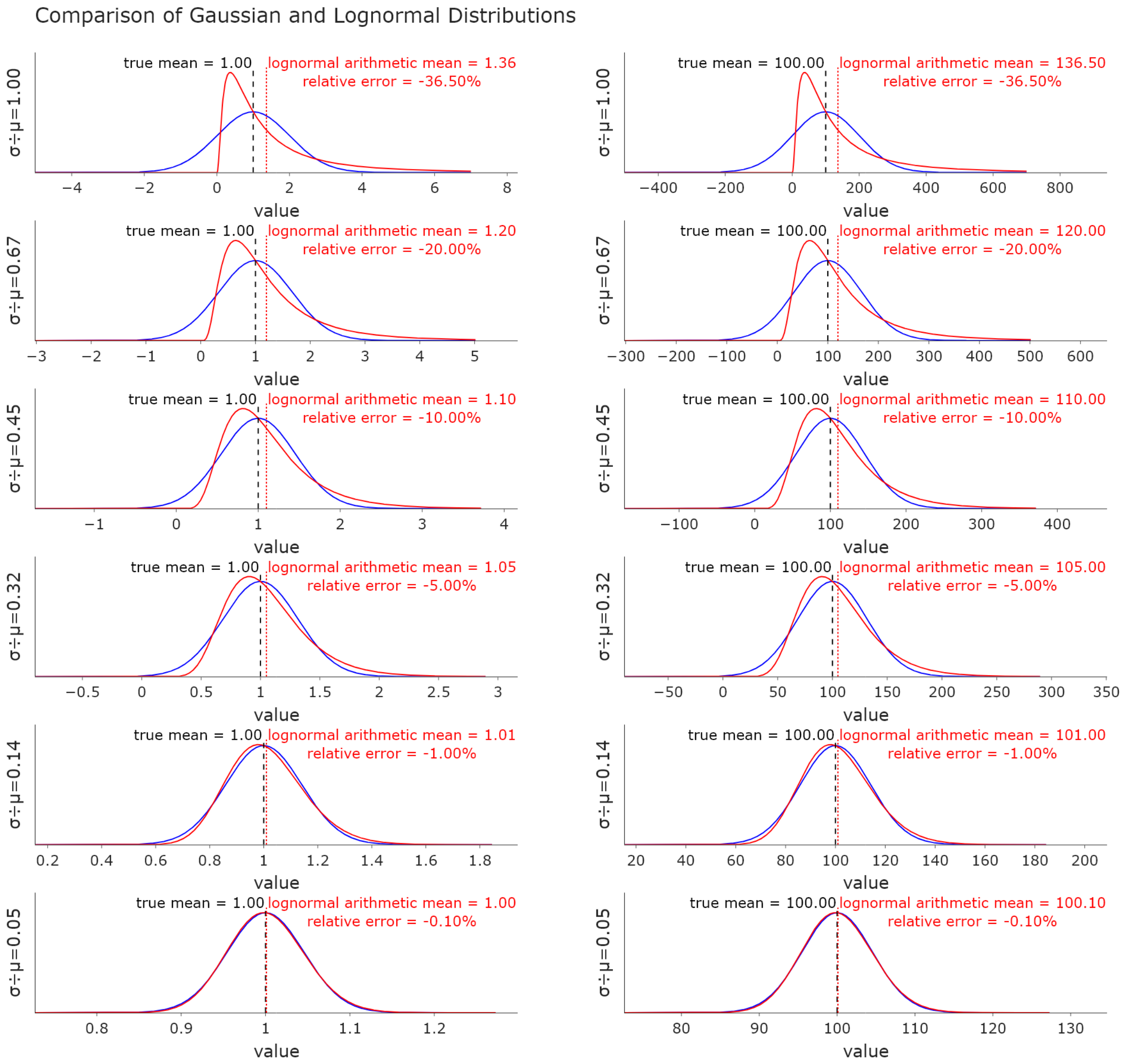

Figure S8: Comparison of lognormal (red) and Gaussian (blue) distributions. The left column are for distributions with means of 1, while the right is for distributions for means of 100. Moving down the columns, the standard deviation of the distributions decreases. Each row shares a common ratio of standard deviation to mean. Also shown is the mean that would be estimated for the lognormal distribution, if the arithmetic mean was used, and the relative error that results from this treatment.

enough (relative to the mean) that the influence this limit is felt, the distribution will be quite different from the Gaussian.

- Errors in mean estimation of 10% occur when the relative standard deviation is 0.45. At this point, the two distributions look significantly different, though when experimental data is used (with its associated noise), it may be hard to tell the difference between samples drawn from these two distributions.

The above focuses on estimation of means. However, the same holds true for estimation of standard deviations—though the error in standard deviations is 1.5 times worse.

Given the above, we think the safest procedure is to always fit a lognormal distribution to the distribution. However, if there is some reason why this is not desired, then one should test the arithmetically estimated standard deviation and mean, and then compute the relative standard deviation ($\frac{\sigma}{\mu}$). If this relative standard deviation is greater than 0.32, we would *strongly* consider if the values obtained by treating the distribution as Gaussian are accurate enough to report.

We also note that the above procedure of calculating the relative standard deviation is to be preferred to 'eyeballing' the distribution to decide if it looks sufficiently Gaussian. Because experimental data will have noise, it could be challenging to determine this by eye. Instead, the relative standard deviation provides a more quantitative guide.

Of course, this discussion does hinge on the researcher understanding the error in parameter estimation they are willing to accept. This, to some extent, also involves understanding both noise and error in measurement. For instance, if the error in the experiment is such that a 10% error in mean estimation is negligible, then this could provide further justification for treating the distribution as Gaussian. However, we once again stress that precision for which mean values can be estimated are often greater than the resolution of the instrument used to take the measurements—because fitting uses data from the *shape* of the distribution, rather than just the peak value. In our experience, we find that proper treatment of the mean from standard TEM can provide precision on at least the 0.1nm scale.

6 Dependence on number of samples

When acquiring data that will be used to estimate the distributions of particles, one must consider the number of measurements to take. The number of samples will determine how precisely the distribution can be estimated.

A rule of thumb that is often used in nanoscience is to measure at least 200 particles. However, it turns out that the precision of estimations will only increase with increasing number of measurements. Thus, the practical reality is always one of trying to balance desired precision against the time required to acquire the increased samples needed to improve the estimations.

Figure S9 illustrates the above point. To generate this figure, we first specify a lognormal distribution with a mean of 2.3 and a standard deviation of 0.8. This is similar to the values found for the smaller particles in the main manuscript. Using this distribution, we then generate a sample of random numbers (based on the lognormal), where the sample size (n_{sample}) varies from 25 to 51200. We then binned this data and fit it to a lognormal, using this fit to extract an estimated mean and standard deviation. The grid of plots in Figure S9a–l illustrate this fitting for each sampling number. Also present are the extracted mean ('center') and standard deviations ('sigma') obtained from the fit. Examining Figure S9 we can see that, as the number of samples increases, the shape of the distribution becomes more clear.

Additionally, for each sample number (i.e., each plot in Figure S9a–l), we repeated the process 100 times (not shown), recording each of the means and standard deviations. Due to the central limit theorem, these parameter estimates are expected to be Gaussian distributed, thus, for each sample size, we calculate the arithmetic mean of the center position, and also provide an estimate of the uncertainty in these values, attained by propagating the standard error from the fits. A plot of these means and standard errors versus the $\log_{10}(n_{sample})$ is shown in Figure S9m, while Figure S9n shows the standard errors versus $\log_{10}(n_{sample})$. It is clear from these final two panels that the mean values attained from the 100 repeat

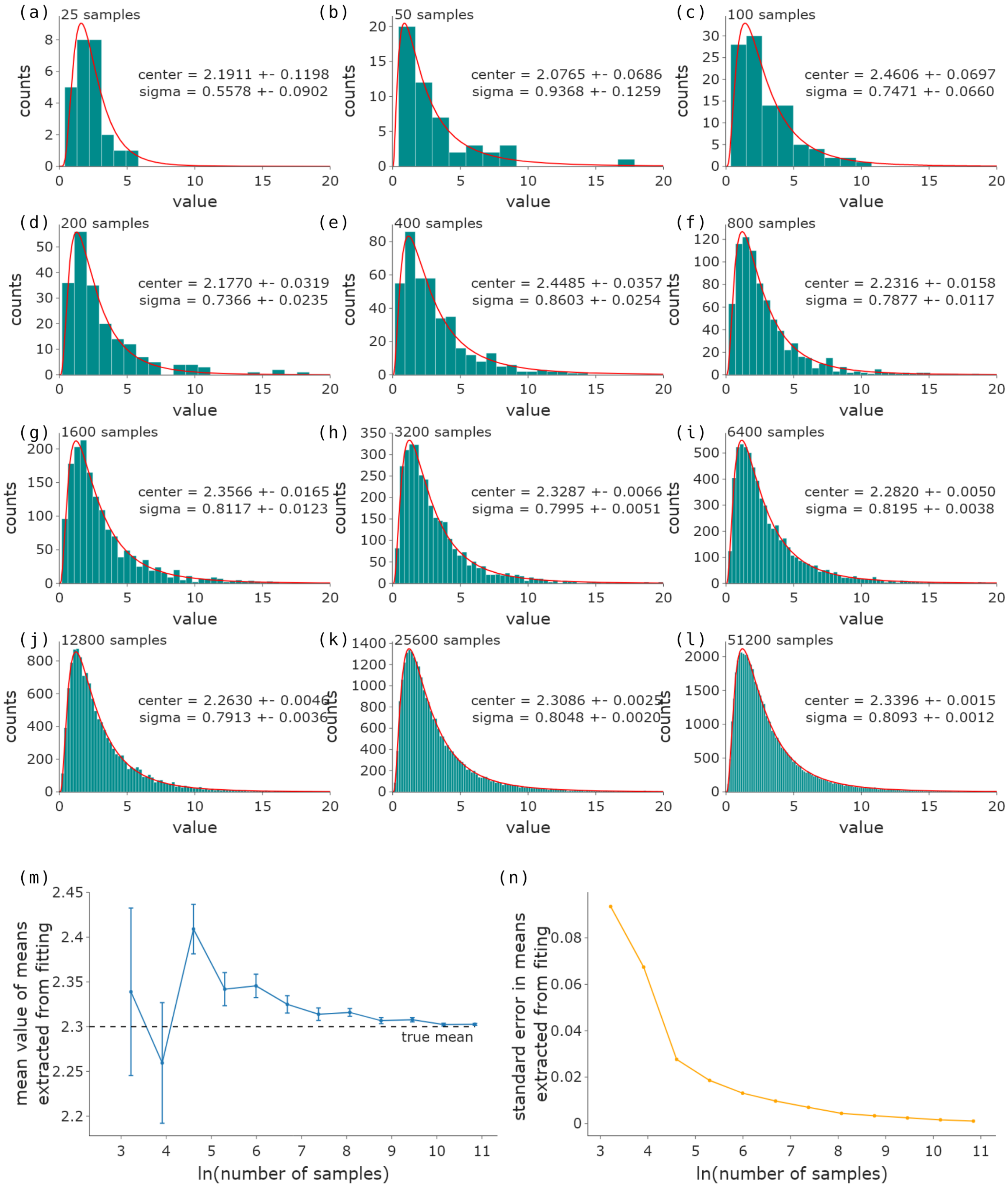

Figure S9: The result of sampling random values from a lognormal distribution with a mean of 2.3 and a standard deviation of 0.8. In a–l, we attain a number of samples given above the plot, bin these samples, and fit the result to a lognormal. We then show this fit (red) and give the extracted mean (center) and standard deviation (sigma) extracted from the fit—together with their uncertainties (standard errors).

sampling/fitting trials approach that of the true mean with increasing sample numbers, and the standard error in the mean estimation decreases monotonically with sample number. Thus, it is clear that higher sample numbers will produce more precise and accurate estimates of the parameters describing the distribution. We note that this data shown is not meant to be fully generalized to all cases, but does give an indication of why 200 samples are often recommended for particle counting—this number has historically represented a good balance between desired precision and time required to analyze TEM images. Of course, if the trainable WEKA approach described in the manuscript is used, then this compromise might move to larger numbers of particles, and therefore greater precision in parameter estimation.
